# Microplastics in freshwater fish from Central European lowland river (Widawa R., SW Poland)

**DOI:** 10.1007/s11356-020-08031-9

**Published:** 2020-02-19

**Authors:** Natalia Kuśmierek, Marcin Popiołek

**Affiliations:** Department of Parasitology, Institute of Genetics and Microbiology, Wrocław University, Przybyszewskiego 63, 51-148 Wrocław, Poland

**Keywords:** Microplastics, Dam reservoir, *Gobio gobio*, *Rutilus rutilus*, Feeding type, Poland

## Abstract

In contrast to marine organisms, the presence of microplastics (MPs) in freshwater animals remains insufficiently studied. The aim of this study was to identify the occurrence of MPs in the digestive tracts of two fish species from a small lowland river (Widawa R., SW Poland). In total, 202 gudgeons and 187 roaches were collected, of which 54.5% and 53.9% had ingested MP-like particles, respectively. Feeding type and behaviour, sex and capture site (above or below the dam reservoir) did not affect the number of fish with MP-like particles.

## Introduction

In recent years, the accumulation of plastic debris in the environment has been considered to be one of the major factors causing a loss in biodiversity and affecting human health (Sutherland et al. 2010; Jiang 2018; Karbalaei et al. 2018). Microplastic (MPs <5 mm in diameter) can enter the environment directly as primary MPs, which were previously used for various purposes, e.g. cosmetic products. In addition, under environmental conditions, larger plastics degrade into smaller items, eventually breaking down into secondary MPs (Cole et al. 2011). So far, marine environments are the best studied ecosystems in the context of MP contamination and to determine the ecological impact of MPs on multiple trophic levels of marine food webs (Avio et al. 2017). Although rivers seem to be the main source of MPs in marine ecosystems, current knowledge about freshwater contamination is scarce (Wagner et al. 2014). Most papers have focused on large rivers that include the Seine, Yangtze and Thames (Zhang et al. 2015; Collard et al. 2018; Horton et al. 2018). However, Sanchez et al. (2014) and Slootmaekers et al. (2019) showed that smaller streams may also contain less intense concentrations of MPs. Fish can ingest MPs in two ways: directly (when consuming prey or attacking items resembling prey) or indirectly (by ingesting prey which itself contains MPs). Recent studies suggest that feeding type and behaviour is related to the MP content in the fish (Sanchez et al. 2014; Mizraji et al. 2017).

Zhang et al. (2015) and Di and Wang (2018) showed that artificial constructions such as dam reservoirs may be a potential area for the accumulation of MPs; however, upstream and downstream differences have not been investigated.

This research aims to determine the occurrence of MPs in the digestive tract of fish from a Central European, small, lowland tributary of the Oder River basin and to compare the level of contamination by MPs in wild fish characterised by different feeding type and behaviour, as well as in fish that live above and below the dam reservoir.

## Materials and methods

**Fig. 1:**
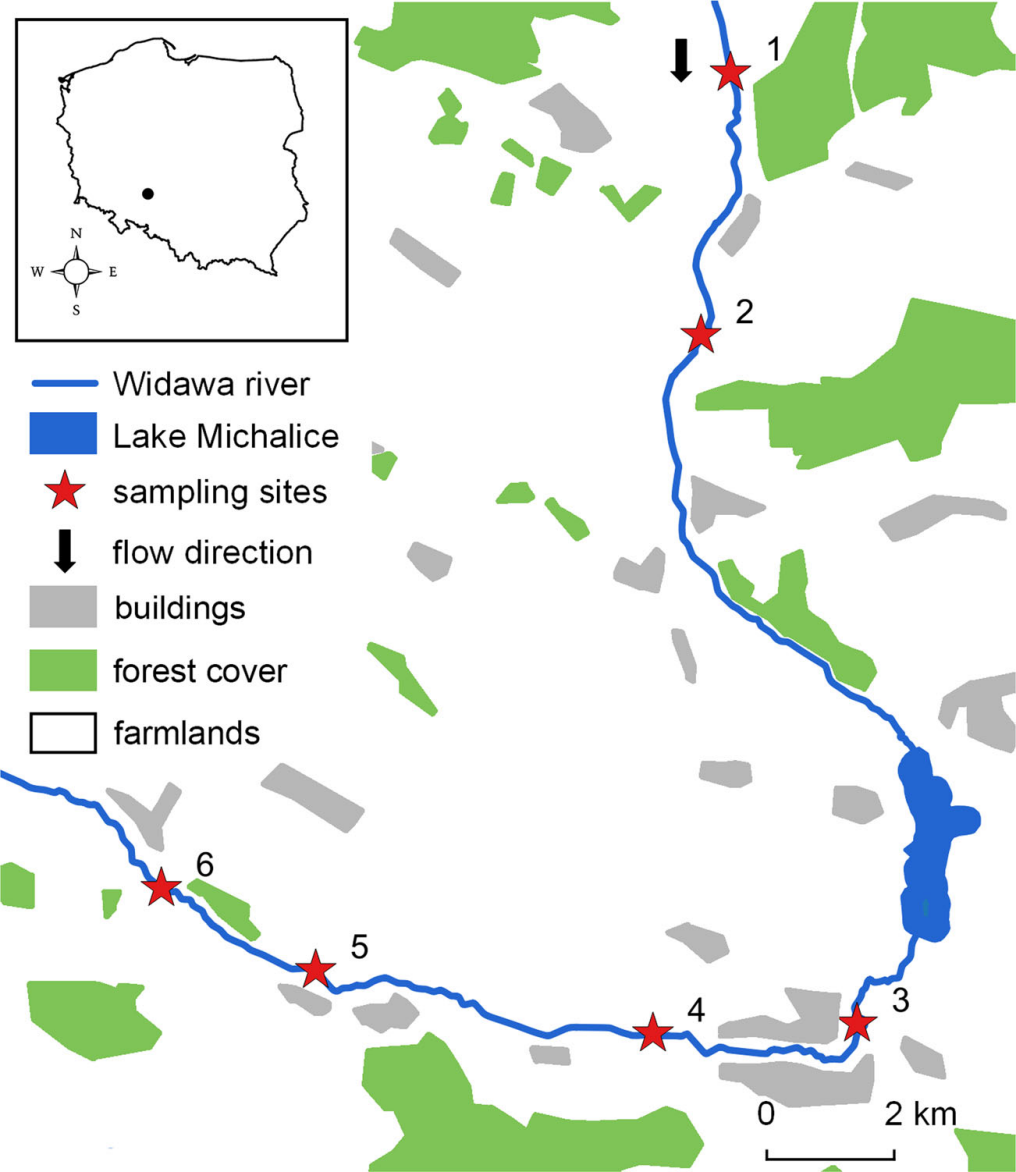
Map showing locations of sampling sites on the Widawa River (SW Poland)

The study was conducted in the Widawa River (a right-bank tributary of the Oder River; river length 109 km, catchment-area 1716 km^2^) in southwest Poland (Fig. 1) between April of 2017 and October of 2018. Six sampling sites were placed along 32 km of the river: two located above and four located below the dam reservoir (Michalice Lake, created in 2001, surface area 95 ha). This area is characterised by a rural landscape with fields, waste lands, small patches of forests, villages and Namysłów town. Gudgeon *Gobio gobio* (benthic, feed on substrate) and roach *Rutilus rutilus* (pelagic, feed in the water column) were electro-fished. After anesthetisation in MS-222, fishes were measured (TL, mm), weighed (W, g) and separately frozen at − 20 °C until further processing.

MP extraction used methods previously described by Foekema et al. (2013) and Dehaut et al. (2016). Before further study, the whole fish digestive tract was screened on petri dishes for internal parasites, which were used for a different (ongoing) project. Afterwards, the entire contents of the petri dish were poured into a glass vessel (150 ml) and flooded with 10% KOH. Contents were incubated for 24 h at 60 °C in a group of 20 samples, along with two control dishes containing 10% KOH solution or distilled water to check for possible human-mediated contamination (no contamination was found in the controls during the MPs extraction). All samples were then searched under a stereomicroscope (Nikon SMZ1000). In order to avoid possible contamination, all liquids were filtered using cellulose filter paper (5 μm porosity) and all equipment was rinsed in ultra-pure water and examined under a stereomicroscope before gut content analysis. In addition, all procedures were carried out in cotton overalls.

Particles were classified as MP-like particles when they had the following characteristics: (1) no cellular or organic structures visible, (2) fibres equally thick at the end, (3) coloured particles were homogenously coloured, (4) fibres were not segmented, or appeared as twisted, flat ribbons, (5) particles were not shiny (Nor and Obbard 2014).

We used a t student test to compare body length and mass between species as well as the mean number of MP-like particles between both species. We also used 2 × 2 contingency tables and Fisher exact tests to compare the proportion of both species that ingested MP-like particles, males and females with MP-like particles (juveniles were excluded from analysis due to small sample size) and fish with MP-like particles above and below the dam reservoir. In this analysis, the threshold for significance was established at *p* < 0.01, after the Bonferroni correction used to avoid Type 1 errors due to multiple comparisons. Statistical analyses were performed using STATISTICA 13.1 software (StatSoft, Poland).

## Results

In total, 389 fish were collected, including 202 gudgeons and 187 roaches. The latter were significantly longer and heavier than gudgeons (t student tests: body length *t* = 8.26, df = 387, *p* < 0.001; body mass *t* = 9.31, df = 387, *p* < 0.001) (Table 1). Of all the sampled fish, 212 (54.5%) had ingested MP-like particles. There were no significant differences between the following: (i) the number of gudgeons and roaches that ingested MP-like particles (Fisher exact test: *p* = 0.84), (ii) sexes with or without MP-like particles in both species (Fisher exact tests: gudgeons *p* = 0.76, roaches *p* = 0.37), (iii) fish with or without MP-like particles collected above and below the dam reservoir (Fisher exact test: *p* = 0.26) (Table 1).

**Table 1:** Number of collected fish and their characteristics

	*Gobio gobio*	*Rutilus rutilus*
Total number of fish	202	187
Mean weight ± SD [g]	14.3 ± 10.6	31.0 ± 23.1
Mean length ± SD [mm]	114.4 ± 23.1	138.0 ± 32.7
Sex (f/m/j)	125/70/7	103/83/1
Number of fish containing MP-like particles (f/m/j)	66/39/4	53/49/1
Maximum number of ingested MP-like particles by fish	8	16
Number of fish without or with MP-like particles		
Above dam reservoir	37/44	44/40
Below dam reservoir	56/65	40/63

In total, 452 MP-like particles were found, including 232 in gudgeon and 220 in roach. In gudgeon, the mean number of MP-like particles ± SD per individual was 1.15 ± 1.65, while in roach it was 1.18 ± 1.89. This difference was not found to be significant (*t* student test: *t* = 0.16, df = 387, *p* = 0.88). In both species, individuals with one MP-like particle were dominant.

**Fig. 2:**
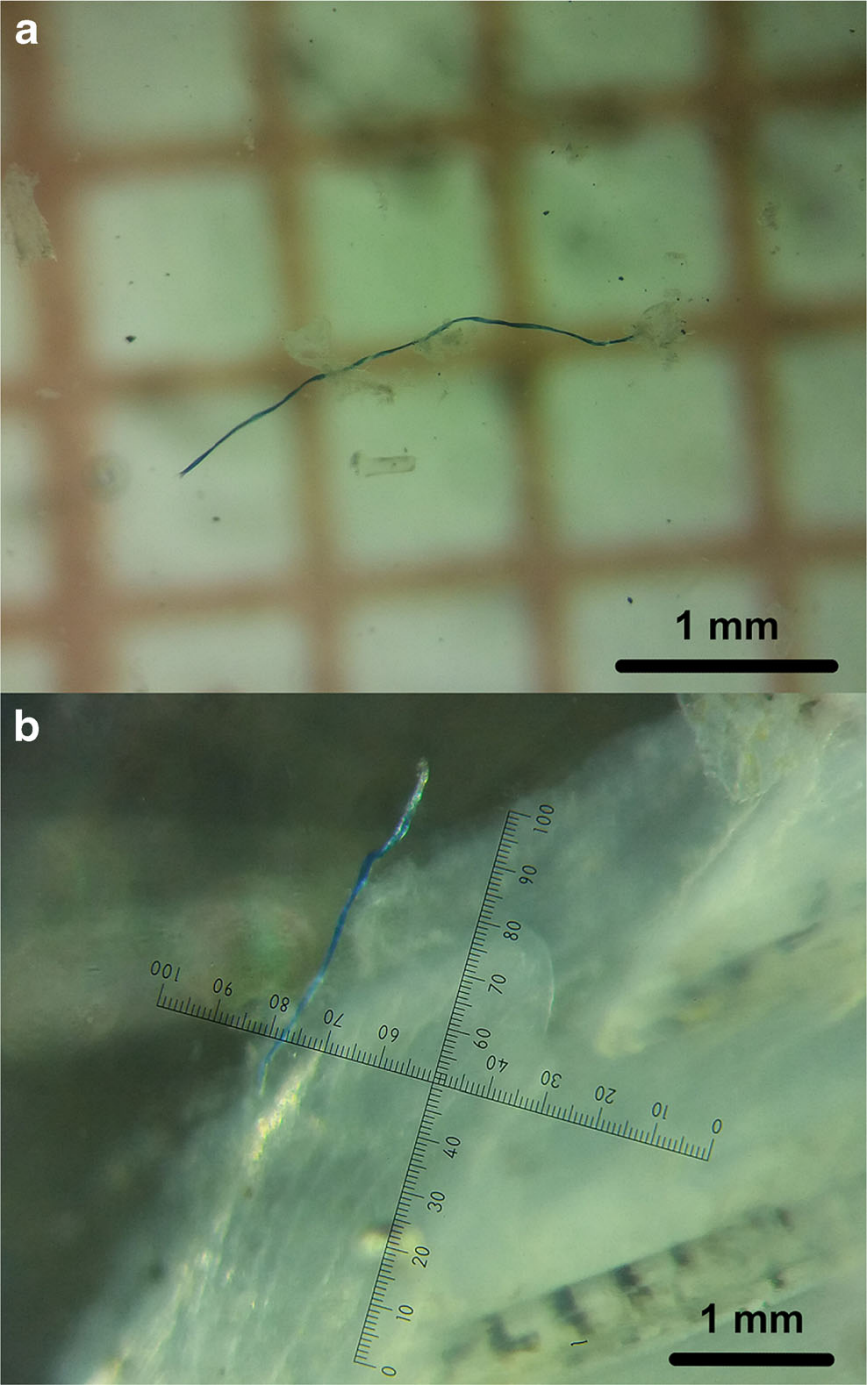
Examples of microplastic-like particles observed: in the digestive tract (**a**), on the fins (**b**)

The majority of particles were fibres (451 examples, accounting for 99.8%) (Fig. 2) followed by only one fragment (0.02%). The length of fibres ranged from 0.5 to 5 mm and the dimension of the fragment was 0.5 × 1 mm. The colours of the identified MP-like particles were diverse (blue, black, transparent, green, red, sky-blue, violet, brown, orange and white).

## Discussion

In our study, MP-like particles were present in fish collected from all study sites and contaminated fish constituted a large percentage of all the collected individuals (more than 50% in both species). This result is higher in comparison with fish collected from other European rivers (e.g. Sanchez et al. 2014; Collard et al. 2018; Roch et al. 2019); however, different methods were used in measuring the occurrence of MPs in fish. Although visual identification of MPs following Nor and Obbard (2014) criteria can be used (e.g. Zhao et al. 2014; Horton et al. 2018), FTIR or Raman spectroscopy is much more reliable. Additionally, although the methods used herein may have omitted the smallest MPs (e.g. less than 0.5 mm), the mean number of MPs per fish was higher than in previous studies (Horton et al. 2018; Roch et al. 2019; Slootmaekers et al. 2019). This confirms that small rivers can be a source of MPs pollution and acts as a corridor connecting lands with larger rivers and further marine ecosystems.

Since we studied only the digestive tract of fish, we are not able to assess how many MPs affect the fish during their entire lifespan. A great deal of MPs is excreted or is accumulated in different organs. However, during dissection, we found at least 20 fish with microfibres tangled in the gills or glued with mucus to the fins (Fig. 2). A high level of MP consumption can negatively affect the health of wild fish due to the accumulation of pathogens or heavy metals, physical injuries, false satiety sensation, etc. Consequently, MPs can lead to disorders that affect growth, reproduction and body conditions (Mizraji et al. 2017; Anbumani and Kakkar 2018; Scherer et al. 2018).

Although previous studies have suggested that feeding ecology can be related to the number of ingested MPs (Lusher et al. 2013; Sanchez et al. 2014; Mizraji et al. 2017), our study did not confirm this hypothesis. No differences between gudgeon and roach can be associated with the low water level in a small river like the Widawa and/or the generally small number of MP-like particles in the studied fish. This indicates that potential differences between species did not affect the results. It can also not be ruled out that the choice of species in this study was unsuitable. While Mizraji et al. (2017) suggested that a higher amount of MP fibres in omnivorous fish (such as the roach) is associated with their wider range of food sources, Sanchez et al. (2014) argued that gudgeons (which eat on the substrate) may be more exposed to MPs than other fish species, including roach. In fact, it is possible that although gudgeons and roach differ in ecology, they are similarly exposed to ingesting MPs.

Our research is the first to show no difference in the accumulation of MP-like particles in fish from above and below the dam reservoir. As dam reservoirs may accumulate the MPs (Zhang et al. 2015), further studies should focus on comparing MP ingestion of different biota living in, above and below dam reservoirs.

Similar to other reports from rivers (e.g. Horton et al. 2018), the main type of MP-like particles in our research was fibres. This kind of MP can be derived from synthetic textiles or hygiene products and constitutes one of the common river pollutants, e.g. over 700,000 fibres can be released per wash into sewage (Napper and Thompson 2016; Pirc et al. 2016).

In summary, our results show that the fish that live in a small river in Central Europe are exposed to the ingestion of MPs. Their behaviour and feeding type, as well as the sampling site (above or below the dam reservoir), do not affect the number of fish with MPs. Further studies should be conducted throughout Central Europe and explore the significance of using different freshwater fish species at various trophic and niche levels.
